# In Situ Synthesis of Doped Bio-Graphenes as Effective Metal-Free Catalysts in Removal of Antibiotics: Effect of Natural Precursor on Doping, Morphology, and Catalytic Activity

**DOI:** 10.3390/molecules28207212

**Published:** 2023-10-22

**Authors:** Maryam Afsharpour, Lugain Radmanesh, Chuanxi Yang

**Affiliations:** 1 Department of Inorganic Chemistry, Chemistry & Chemical Engineering Research Center of Iran, Tehran 14335-186, Iran; 2 School of Environmental and Municipal Engineering, Qingdao University of Technology, Qingdao 266520, China

**Keywords:** bio-graphene, natural gum, metal-free catalyst, antibiotic

## Abstract

Wastewater contaminated with antibiotics is a major environmental challenge. The oxidation process is one of the most common and effective ways to remove these pollutants. The use of metal-free, green, and inexpensive catalysts can be a good alternative to metal-containing photocatalysts in environmental applications. We developed here the green synthesis of bio-graphenes by using natural precursors (Xanthan, Chitosan, Boswellia, Tragacanth). The use of these precursors can act as templates to create 3D doped graphene structures with special morphology. Also, this method is a simple method for in situ synthesis of doped graphenes. The elements present in the natural biopolymers (N) and other elements in the natural composition (P, S) are easily placed in the graphene structure and improve the catalytic activity due to the structural defects, surface charges, increased electron transfers, and high absorption. The results have shown that the hollow cubic Chitosan-derived graphene has shown the best performance due to the doping of N, S, and P. The Boswellia-derived graphene shows the highest surface area but a lower catalytic performance, which indicates the more effective role of doping in the catalytic activity. In this mechanism, O_2_ dissolved in water absorbs onto the positively charged C adjacent to N dopants to create oxygenated radicals, which enables the degradation of antibiotic molecules. Light irradiation increases the amount of radicals and rate of antibiotic removal.

## 1. Introduction

In recent years, wastewater contaminated with pharmaceuticals has been one of the most important environmental concerns, which has become an emerging environmental challenge [1,2,3,4,5]. Every year, many antibiotics which are used to increase the health quality of humans, are released into the environment. Since the conventional wastewater treatment methods used by the pharmaceutical industry are not able to completely remove antibiotics, therefore, the wastewater contains a significant amount of antibiotics. In addition, the unmetabolized antibiotics released by humans and animals have led to the large amounts of antibiotics in municipal and livestock wastewaters. These antibiotics in wastewater lead to the challenges of antibiotic resistance, which is a major environmental problem threating the human health and safety of other terrestrial and aquatic organisms [5,6,7,8]. Currently, several physical, chemical, and biological technologies have been developed to remove antibiotics from wastewater [9]. Physical methods include filtration, membrane, absorption, coagulation, and sedimentation, which are based on electric attraction, van der Waals forces, gravity, and using physical barriers [10,11]. These methods need post-treatment for accumulated pharmaceutical contaminants. In biological methods, microorganisms are used to break down organic pollutants using cellular processes and convert them into simple materials and biomass [12,13]. These methods in a traditional way are also ineffective. Catalytic, photocatalytic, and electrocatalytic processes, ion exchange, and oxidation are the chemical processes used for wastewater treatment [14,15,16,17]. Among these methods, advanced oxidation processes (AOPs) are the well-known and effective methods for the removal of various organic pollutants such as dyes, antibiotics, pesticides, etc. [18,19,20,21,22,23,24]. AOPs are involved in the in situ generation of sufficient oxygenated radicals for interaction with the organic pollutants in the wastewater medium such as Fenton processes, ozonation, wet air oxidation, photochemical, and electrochemical. Metal oxide semiconductors such as TiO_2_, ZnS, ZnO, WO_3_, SnO_2_, BiVO_4_, AgBr, and MOFs are the largest group of these catalysts that can fully destroy organic pollutants via catalytic, photocatalytic, or electrophotocatalytic processes [24,25,26,27,28,29,30]. Another category of AOP catalysts are metal-free catalysts. Many metal-containing catalysts are expensive, toxic, or ineffective. Therefore, the design of low-cost, high-performance metal-free catalysts is significant. From this category, we can mention carbon nitrides (g-C_3_N_4_) [31,32,33] and silicon carbides (SiC) [34], which are more attractive due to their low cost, high stability, good chemical stability, and high thermal conductivity, as well as being environmentally friendly, compared to metal-containing catalysts. As such, they have been widely used in water remediation and antibiotics removal. 

The high recombination rate of photogenerated electron-hole and wide band-gap is the disadvantage of these catalysts. To overcome it, these catalysts are modified with metal or metal oxides to improve their catalytic activities. These metals are expensive, and also, the leaching of these metal ions might cause secondary pollution. Thus, it is challenging to find low-cost and high-effective co-catalysts to modify the efficiency of metal-free catalysts such as g-C_3_N_4_. Using other metal-free catalysts, graphenes, or doping these compounds with other non-metal elements can be an effective and cheap way to improve the catalytic properties of these compounds. The combination of g-C_3_N_4_ with SiC and siligraphene (g-SiC) has created effective photocatalysts in the removal of dyes and antibiotics [34,35,36]. 

Although carbon nanostructures such as graphene are often used as adsorbents to remove pollutants, these carbon compounds are used to increase the surface area of other photocatalysts to subsequently increase the absorption and improve the catalytic properties [37]. Also, the doped structures of these compounds can be used as good oxidation catalysts [38,39,40,41,42]. Kang et al. [38] and Wu et al. [39] reported the degradation of antibiotics in the presence of N-doped graphene for the activation of peroxymonosulfate. 

In this study, doped bio-graphenes as the metal-free green catalysts were in situ synthesized using natural precursors. The use of natural resources can be a way to produce cheap, safe, and environmentally friendly compounds with a porous structure and special morphology. In this study, the effect of natural precursors on morphology, surface area, doping, and band levels was evaluated here. The doped structures were then tested for degradation of antibiotics via an advanced oxidation mechanism, and different structural parameters were investigated. In this research, only saturated oxygen in water (air bubbling) was used, and no oxidants were added to the system. However, the effect of oxygen and light has also been investigated in accelerating the degradation process in the removal of antibiotics.

## 2. Results and Discussion

### 2.1. Characterization of Synthesized Bio-Graphenes

Carbon-based materials which are synthesized from natural materials (biochars or bio-graphenes) are of particular importance due to the easy, cheap, and green synthesis method. The use of natural precursors can act as a template and create a structure with special porosity and morphology. Here we have chosen natural gums as precursors. These natural polymers can create a unique polymeric network to produce the 3D graphene structures. The porosity and morphology of these graphenic structures are directly dependent on the natural precursors.

**Figure 1:**
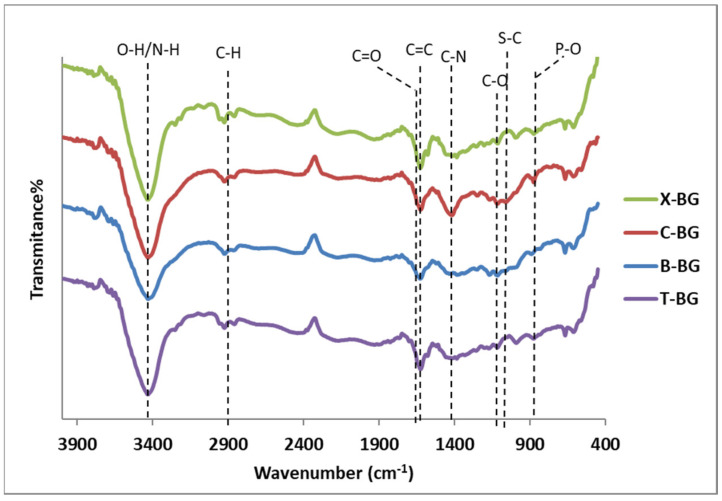
FT-IR spectra of synthesized bio-graphenes.

Figure 1 shows the FT-IR spectra of synthesized bio-graphenes. In all of the samples, the stretching vibrations of the C=C, C-O, and C=O bonds were observed at about 1115, 1630, and 1720 cm^−1^. Also, the broad bands at about 3300–3600 cm^−1^ were related to stretching vibrations of O-H and N-H bonds, and the absorption bands at about 2925 and 2895 cm^−1^ indicate the symmetric and asymmetric C-H vibrations. In the X-BG, C-BG, and T-BG samples, which show the N-doping, the C-N absorption bands appeared at 1425 cm^−1^, and the presence of P and S dopants in the C-BG sample was observed at 895 cm^−1^ and 1080 cm^−1^, indicating the S-C and P-O bonds in this doped graphene.

**Figure 2:**
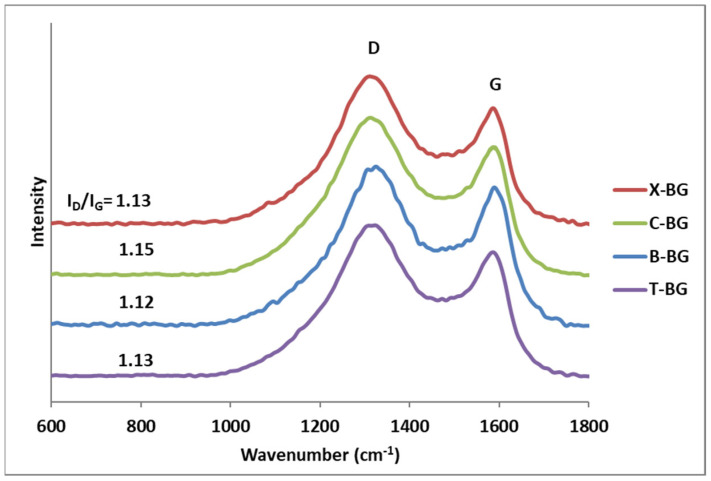
Raman spectra of synthesized bio-graphenes.

**Figure 3:**
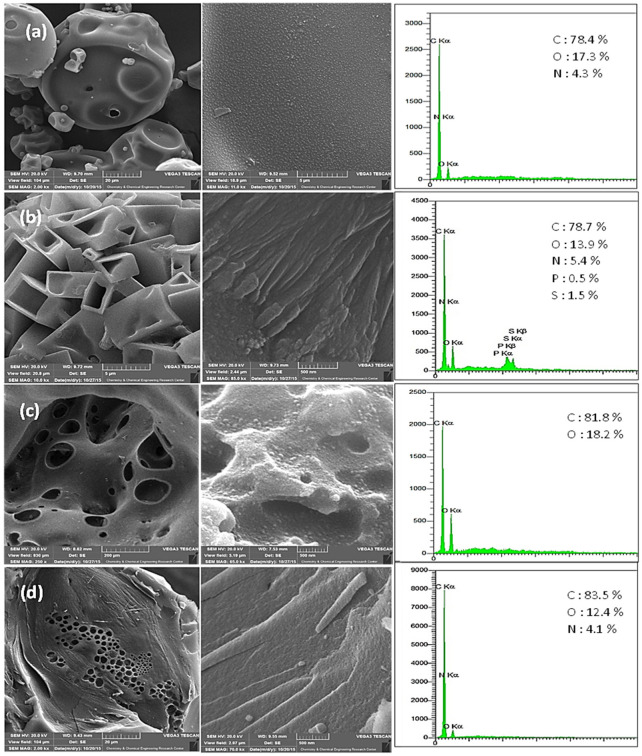
SEM images and EDX spectra of (**a**) X-BG; (**b**) C-BG; (**c**) B-BG; and (**d**) T-BG.

Figure 2 shows the Raman spectra of synthesized bio-graphenes. Two characteristic graphene bands of D and G were observed for all samples at about 1584 and 1313 cm^−1^, respectively. The first-order G band is the primary mode which represents the sp^2^ planar configuration of carbon and the second-order D band is the disorder band which represents a ring mode of carbon adjacent to a defect. So, the intensity of the D band was increased by increasing the defects in the graphene structure. The doping of heteroatoms increased the degree of disorder and the intensity ratio of D to G bands (I_D_/I_G_) was increased. As shown in Figure 3, the highest I_D_/I_G_ ratio is related to the C-BG graphene which was derived from Chitosan. It indicates the higher defects in the C-BG graphene structure due to the doping of N, P, and S. The lowest I_D_/I_G_ ratio is related to the B-BG which shows the graphene structure with no element doping.

Figure 3 shows the SEM images of bio-graphenes synthesized from different natural sources. As can be seen, several morphologies have been created using different natural precursors. Figure 3a shows the hollow sphere structure obtained from natural Xanthan. This compound is produced from the fermentation process of single sugars by *Xanthomonas campestris* bacteria. The EDX results show the N-doped graphene structure for the compound, which is caused by the presence of nitrogen compounds in the original natural composition. Na, K, Ca, and Mg elements have been removed in the acid washing step, which themselves are effective in creating porosity in the structure. Figure 3b shows the hollow cubic structure for bio-graphene derived from natural Chitosan. Chitosan is a linear polysaccharide which is made from the chitin shells of shrimp and other crustaceans. The presence of amino groups in the structure of this compound has created a N-doped structure in the obtained graphene. The presence of P and S elements in this structure indicates the presence of compounds containing these elements in the original natural composition, which has led to the creation of N, P, and S-doped graphenes. Figure 3c presents the SEM image of bio-graphene derived from Boswellia resin. Boswellia is a sticky herbal extract made from the Boswellia tree. This resin produces a porous bio-graphene resulting from the 3D network created by this polymer. SEM images of bio-graphenes derived from Tragacanth gum are presented in Figure 3d. Tragacanth is a natural gum obtained from Middle Eastern legume species. As can be seen, this compound (T-BG bio-graphene) shows a layered porous structure in which nitrogen is doped. 

**Figure 4:**
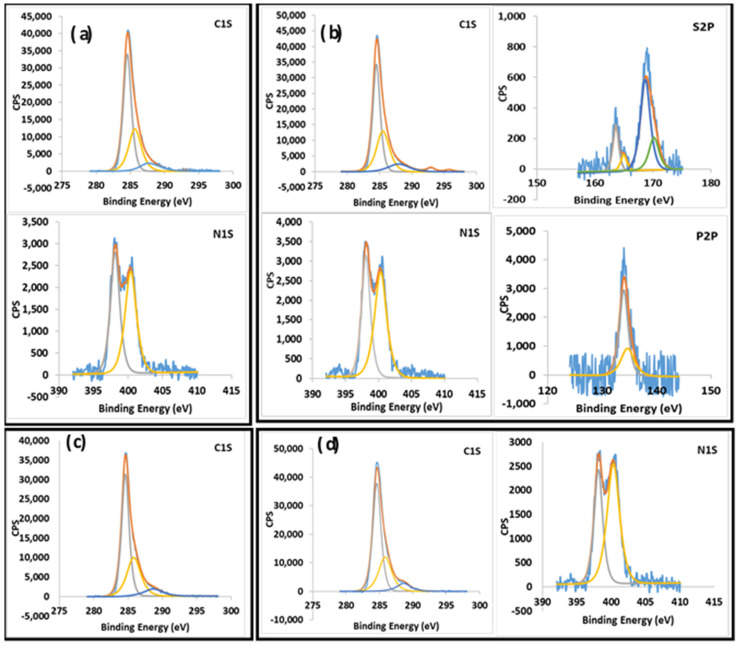
XPS analysis of synthesized bio-graphenes (**a**) X-BG; (**b**) C-BG; (**c**) B-BG; and (**d**) T-BG.

To confirm the placement of N, P, and S in the graphene structures, XPS analysis was conducted (Figure 4). In all of the samples, the C1s peaks are deconvoluted into three bands at about 284.5, 285.5, and 289.0 eV, which corresponded to C-C, C-O/C-N, and C=O species, respectively. Samples X-BG, C-BG, and T-BG show the doping of nitrogen in the graphene structures. The pyridinic N and pyrrolic N (398.5, and 400.5 eV) are the nitrogen species which are characterized in all of the samples. The amount of pyridinic N is higher than the pyrrolic N species in the X-BG and C-BG samples. In the T-BG sample, the pyrrolic N is dominant. Previous results show that these nitrogen species are effective in catalytic oxidative reactions [43,44,45,46]. The amounts of nitrogen were measured as 4.5, 5.1, and 4 for X-BG, C-BG, and T-BG, respectively, which is in agreement with the EDX analysis. The presence of P and S in the B-BG structure was also confirmed by XPS analysis (Figure 4b). The results show the bonding of phosphor with oxygen (P-O) at 134.2 and 134.9 eV and the bonding of sulfur with carbon (S-C) and oxygen (C-SO_x_-C) at 168.9 eV and 170.6 eV, respectively. The amount of P and S obtained was 0.3 and 1.4, respectively.

**Figure 5:**
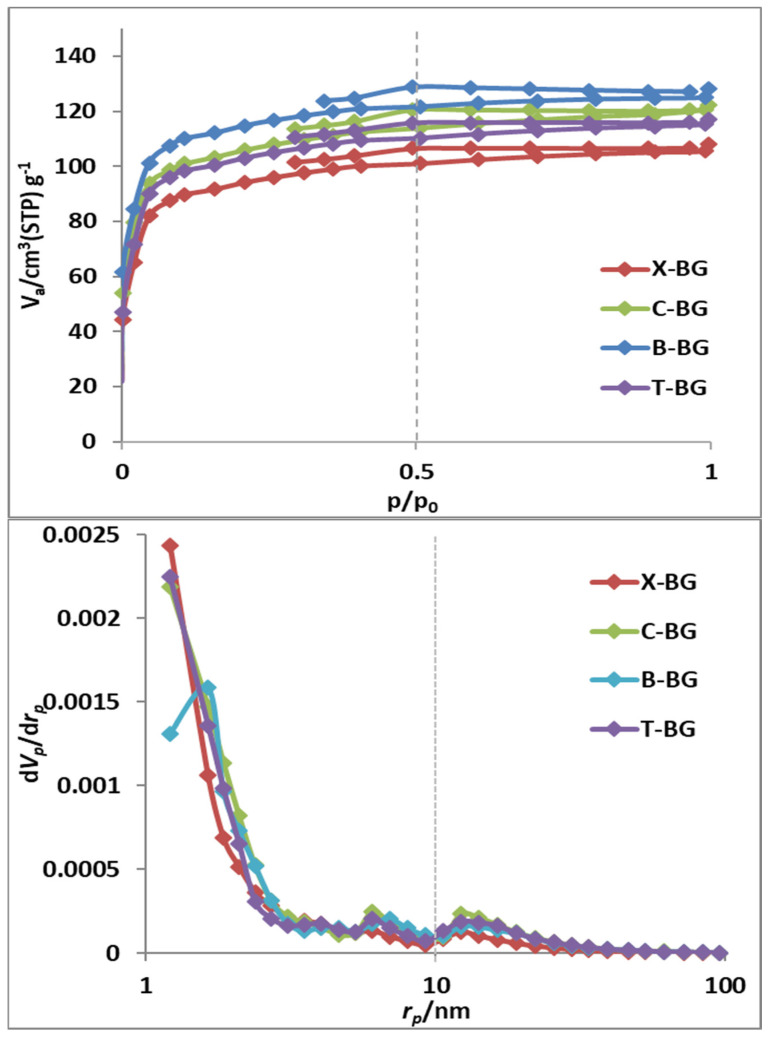
N_2_ adsorption/desorption isotherms and pore size distribution curves of synthesized bio-graphenes.

Since the amount of adsorption is related to the porosity of the substrate, the porosities of synthesized bio-graphenes are discussed in Figure 5. As shown in the N_2_ adsorption/desorption isotherms and pore size distribution curves, the highest surface area with different pore distribution was observed in the B-BG sample. This observed increase in surface area with different pores is dependent on its initial precursor, creating a porous structure with special morphology. After B-BG, the largest surface area is related to the C-BG sample, which has the highest amount of dopants. The structure of the primary polymer precursor and the presence of various dopants in the final graphene structure have created a hollow cubic structure and increased surface area in this compound. As shown in Figure 4, the X-BG and T-BG samples show almost the same surface area. These two samples show more similar nitrogen amounts and morphologies.

Next, the role of doped porous graphene structures obtained from natural sources in the catalytic removal of antibiotics will be investigated.

### 2.2. Catalytic Study

In order to investigate the catalytic properties of synthesized bio-graphene, the removal of antibiotics from wastewater was investigated. Tetracycline (TCL), ciprofloxacin (CIP), and amoxicillin (AMX) were tested as the model antibiotics and the removal efficiency of the synthesized catalysts was evaluated in the presence of air. The effect of oxygen and light on accelerating the process was also investigated.

**Figure 6:**
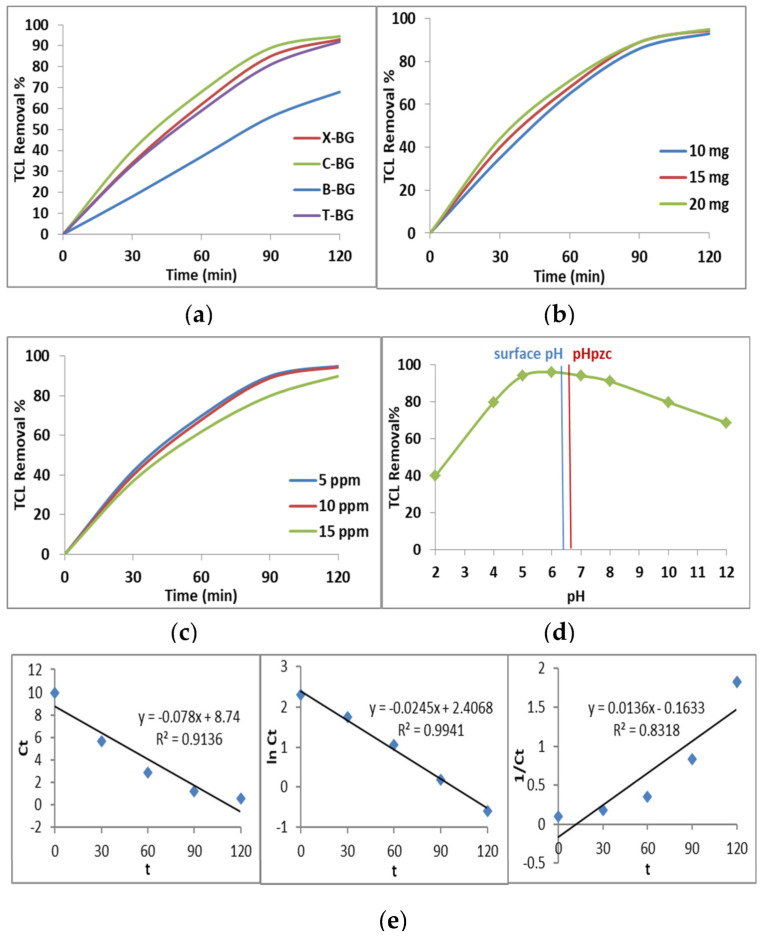
Catalytic efficiency of synthesized bio-graphenes in the removal of TCL (**a**); effect of different amount of C-BG catalyst (**b**); effect of different TCL concentrations (**c**); effect of pH (**d**); and kinetic model of TCL removal (**e**).

Figure 6a shows the catalytic removal of tetracycline (TCL) using bio-graphenes derived from different natural precursors. As can be seen in Figure 6, the highest amount of TCL removal is related to the C-BG sample, in which N, P, and S elements are doped, resulting in the role of dopants improving catalytic properties. The X-BG and T-BG samples also show higher catalytic activities than the B-BG catalyst, although B-BG has the largest surface area. This result shows that the effect of doping was far greater than the effect of surface area in catalytic activity. The X-BG and T-BG samples, which have almost equal amounts of nitrogen and surface area and similar morphology, show similar catalytic results.

Figure 6b–d shows the optimization of the catalytic conditions of C-BG in the removal of TCL. The results show that by increasing the amount of catalyst to 15 mg, the catalytic activity increased, but the further increase in the amount of the catalyst only increased the rate of reaction (Figure 6b). Figure 6c shows that the best result in the removal of TCL is related to lower concentrations of this antibiotic; by increasing the concentration of the antibiotic, the amount of removal decreases slightly.

Figure 6d displays the effect of pH on the catalytic activity of C-BG on TCL removal. As we know, the ionic properties of the antibiotic molecule and the surface of the catalyst are effective on the absorption efficiency. The surface charge of the catalyst is negative at a pH higher than pH_zpc_ (pH of zero point charge) and can adsorb the positive molecules. At a pH lower than pH_zpc_, the surface of the catalyst is positive and can adsorb the negative molecules. The pH_zpc_ of C-BG is calculated as 6.7, which is higher than the surface pH (6.4), so it can adsorb the negative molecules. As shown in Figure 6d, the removal of TCL increased via increasing the pH and reached its maximum at pH 4–7; then, a decrease occurred at pH > 7.

TCL has several ionizable functional groups such as tricarbonyl amide, dimethylammonium, and phenol diketone. The cation species of H_2_TC^+^ was observed at pH < 3.3, zwitterionic species of H_2_TC^0^ was formed at pH 3.3–7.7, and anion species of HTC^−^/TC^2−^ was observed at pH > 7.7 [47,48].

Due to the electronegativity difference between C and N, in doped structures, carbon atoms have a partial positive charge and nitrogen atoms have a partial negative charge. At a pH of 4–7, the surface of the catalyst has a pH_zpc_ of 6.7 and is positive in this pH, and zwitterionic H_2_TC^0^ is the main species of TCL, therefore, TCL can adsorb onto the surface of the catalyst via the interaction between the negatively charged tricarbonyl amide groups of TCL and the positive centers (C adjacent to N, P, and S) in the surface of the catalyst. Thus, the best catalytic result of the C-BG catalyst is obtained at a pH of 5–7, which is lower than pH_zpc_ (6.7). When the pH decreases to a pH lower than 4, the TCL removal reduces due to the repulsion between the cationic species of TCL and the positively charged catalyst (pH < pH_zpc_). By increasing the pH to more than the pH_zpc_ of the catalyst, the electrostatic repulsion between the negatively charged catalyst and anionic TCL decreases the removal efficiency (Figure 6b) 

Figure 6e exhibits the rate of adsorption/degradation of tetracycline on the surface of the C-BG catalyst to obtain a better understanding of this process. Three different fittings of kinetic models, pseudo-zero-order, pseudo-first-order, and pseudo-second-order, are reported in Figure 6e for TCL removal. As can be seen, the highest correlation coefficient (R^2^) of tetracycline adsorption/degradation was related to the pseudo-second-order kinetic model, so the antibiotic degradation process followed this model. 

**Figure 7:**
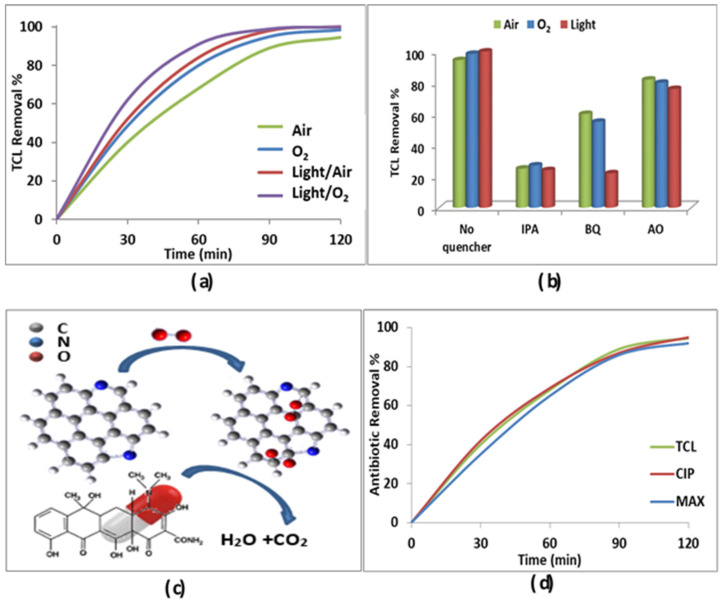
Catalytic performances of C-BG in the removal of TCL in the presence of air, O_2_, and light (**a**); quenching test in TCL removal in the presence of air, O_2_, and light (**b**); mechanism of TCL removal (**c**); and catalytic performances of C-BG in the removal of different antibiotics (**d**).

To investigate the catalytic removal mechanism, the reaction was also performed in the presence of O_2_ and light, and the radicals participating in the reaction were identified. Figure 7a shows the results of catalytic activity in different conditions. As can be seen, by using O_2_ gas instead of air, the rate of reaction and yield of TCL removal increases, which shows that oxygen is involved in the reaction mechanism. 

Figure 7b shows the quenching test results. In both conditions, the presence of air and O_2_, the formation of oxygenated radicals was observed (Figure 7b). In the air condition, hydroxyl (^•^OH) radical is the dominant active species, while in the presence of O_2_, the role of the ^•^O_2_^−^ radical increases. By irradiating light to the system, the rate of reaction and the yield of TCL removal increases (Figure 7a), indicating an increase in the production of oxygenated radicals. According to Figure 7b, the active radical in this system is the ^•^O_2_^−^ radical.

In Figure 7c, the mechanism of TCL removal was displayed. In this mechanism, the oxygen molecules dissolved in water (saturated by air bubbling) adsorb onto the positive carbon atoms adjacent to nitrogen atoms [45]. Doping the nitrogen onto the graphene structure creates the positively charged carbon atoms, which are induced by the charge polarization of nitrogen atoms. These positively charged C atoms make the active sites adsorb the oxygen molecules to produce the oxygenated radicals. As shown in Figure 7c, O_2_ and H_2_O molecules can easily chemisorb on doped graphenes via the bonding of O atoms to positive carbon sites. After chemisorption of O_2_ on the surface of the catalyst, the O-O bond is dissociated and different oxygenated radicals are produced [21]. The proton of chemisorbed water molecules is also dissociated and OH groups are formed by connecting to dissociated oxygen molecules. The produced oxygen-containing radicals can proceed the degradation of TCL via oxidation reaction [20,21,45]. P and S dopants also have the same function as nitrogen [35]. The B-BG sample which has the graphene structure with no dopants can only remove the TCL via the adsorption mechanism.

To present the efficiency of these doped bio-graphenes at removing the different antibiotics, the removal of ciprofloxacin (CIP) and amoxicillin (AMX) were also investigated (Figure 7d). The results show good catalytic activity of C-BG in the removal of various antibiotics. This result can be a confirmation of the efficiency of these catalysts in removing different types of antibiotic pollutants.

**Figure 8:**
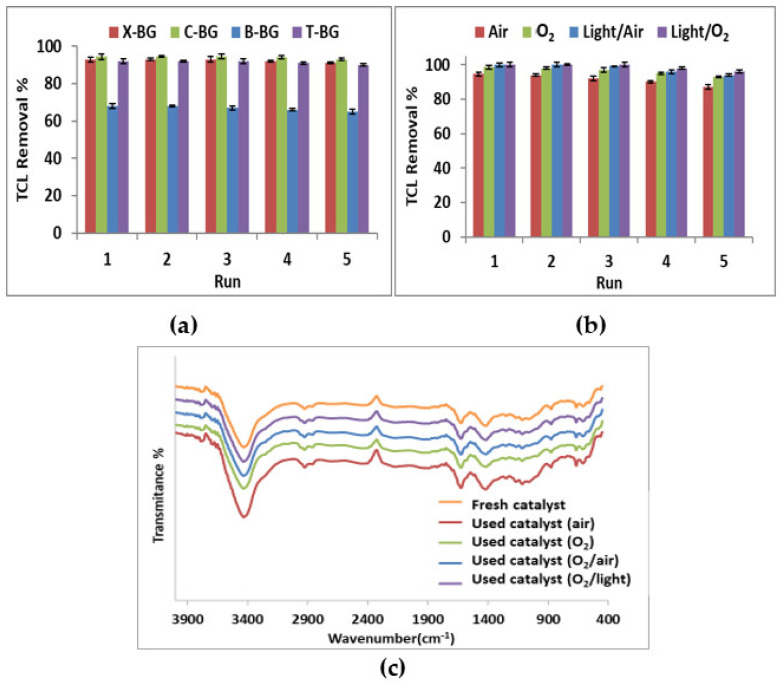
Recovery of catalysts in TCL removal under air bubbling after washing the catalysts (**a**); recovery of C-BG catalyst without washing at different conditions (**b**); and FT-IR spectra of fresh and used C-BG catalysts in different catalytic reaction conditions (**c**).

Figure 8 shows the stability of these catalysts as one of the characteristics of a good catalyst. In order to test the stability of these catalysts, the recovery was performed in two cases. In the first case, after the completion of the reaction, the catalysts were filtered and washed well with water to separate the absorbed drugs from the surface of the catalysts. Then, the catalysts were dried and used again in the tetracycline removal reaction. In the second case, the reaction was carried out continuously. And after the completion of the reaction, the catalysts were used in the next reaction without washing. If absorption had happened on the surface of the catalyst or if the percentage of degradation was low, the surface of the catalyst was covered with antibiotics and the continuation of the process would result in reduced activity. But if complete degradation had occurred, the catalyst surface was empty and ready for the next reaction. To prove this, FT-IR spectra were also prepared from the used catalysts to prove the absence of the drug on their surface.

Figure 8a shows the recovery of catalysts after washing them with water in the removal of TCL under air bubbling. The results show slight decreases in their catalytic properties after five runs, confirming the structural stability of these compounds. Figure 8b displays the stability of the C-BG catalyst in a continuous system, and the recovery of the C-BG catalyst was evaluated at different reaction conditions without washing the used catalyst. The results show a decrease in catalytic activity after three runs when only air entered to system. Whereas, in the presence of oxygen and light, no decrease was observed even after five runs. These results show that air can promote the TCL degradation process well. However, the lower rate of removal and the presence of small amounts of antibiotics on the surface of the catalyst cause a slight reduction in removal to be observed in subsequent runs, which is solved by washing the catalyst to prevent the reduction in activity (Figure 8a). But, when oxygen and light were used, complete decomposition occurred, which is a possibility of using this catalyst in a continuous system with the need to wash the catalyst in more runs.

The FT-IR of the fresh C-BG catalyst and its used ones at different conditions are shown in Figure 8c. As shown, no additional peaks related to TCL are observed in the used catalysts (without washing), which indicates the absence of absorbed antibiotic molecules on the catalyst surface and confirms the removal of antibiotics based on the process of degradation and not absorption.

## 3. Experimental

### 3.1. Materials and Methods

Natural precursors were used for the synthesis of bio-graphenes. Xanthan, Chitosan, Boswellia, and Tragacanth gums were purchased from a local shop with 70–80% purity and used after washing with water to remove the dust. Tetracycline (TCL), ciprofloxacin (CIP), and amoxicillin (AMX) were selected as the model antibiotics to test the removal efficiency of synthesized bio-graphenes.

Scanning electron microscopy with energy dispersive X-ray spectroscopy (SEM/EDX) was used for surface analysis by a TESCAN, VEGA3 microscope, Czech Republic. FT-IR spectra were taken from a Bruker, Vector spectrometer, Germany. Raman spectra were obtained from Bruker (Billerica, MA, USA), Senterra micro-Raman, Stuttgart, Germany. ESCALAB 250Xi Thermo Scientific system (Waltham, MA, USA) was used for XPS analysis by using a spectrometer with MgKα = 1253.6 eV. N_2_ adsorption/desorption isotherms and the pore size distributions were recorded by a Belsorp mini instrument (Sapporo, Japan). Photocatalytic tests were measured by a Perkin-Elmer, Lambda-35 UV-Vis spectrometer (Waltham, MA, USA).

### 3.2. Synthesis of Bio-Graphenes

5 g of each natural precursor (Xanthan, Chitosan, Boswellia, or Tragacanth gum) was dissolved in 50 mL water at 50 °C. The solution was stirred until clear gel was obtained. The solutions were filtered to remove the impurities. Then, the obtained gels were dried at 80 °C for 12 h, and the resulting powders were transferred to a crucible and heated at 750 °C with the rate of 5°/min for 1 h at N_2_ atmosphere. The obtained bio-graphenes were washed with diluted HCl to remove the metals (Ca, Mg, K, and Na species). The bio-graphenes were obtained from Xanthan, Chitosan, Boswellia, and Tragacanth gums named X-BG, C-BG, T-BG, and B-BG, respectively.

### 3.3. Catalytic Study

To investigate the performance of synthesized bio-graphenes in the catalytic removal of antibiotics, a certain amount of doped graphenes was added to 10 mL of tetracycline (TCL) solution with different concentrations (5, 10, and 15 ppm). The system was saturated with oxygen by air bubbling during stirring. To optimize the photocatalytic reaction, different amounts of graphene catalyst (10, 15, and 20 mg) were tested for degradation of TCL at a pH of 2–12. First, all of the samples were stirred for 10 min to reach the equilibrium and then, the removal of TCL was measured in the presence of dissolved oxygen. After every 10 min, TCL concentration was measured by UV-Vis spectroscopy at 357 nm using a calibration curve. The calibration curve of TCL standard solutions displays the concentration of TCL dependence of absorbance at 357 nm (maximum adsorption wavelength of TCL). First, the stock solution of TCL was prepared in 50 ppm concentration. Then, 5 series of standard solutions were prepared from this stock solution by means of dilution to 25, 12.5, 6.25, 3.125, and 1.562 ppm. Then, the calibration curve was plotted (R^2^ = 0.9996), and the unknown concentration was measured according to the absorbance at 327 nm. The TCL removal % was determined using the following equation:

$$\mathrm{Removal}\;\%=\left[\frac{\left(C_{0}-C_{t}\right)}{C_{0}}\right]\times 100\%$$

where C_0_ and C_t_ are the initial and equilibrium TCL concentrations, respectively.

In order to investigate the role of oxygen and light, the above process was repeated in the presence of O_2_ gas and under visible light irradiation. To investigate the reaction mechanism and determine the active radicals, the quenching tests were performed via using 2 Mm of isopropanol (IPA) as the scavenger of hydroxyl radical (^•^OH), 1,4-benzoquinone (BQ) as the scavenger of superoxide radical anions (^•^O_2_^−^), and ammonium oxalate (AO) as the hole (h^+^) scavenger.

In order to check the ability of the mentioned catalysts to remove all kinds of antibiotics, catalytic tests were also performed for two other antibiotics (ciprofloxacin (CIP), and amoxicillin (AMX)).

**Scheme 1:**
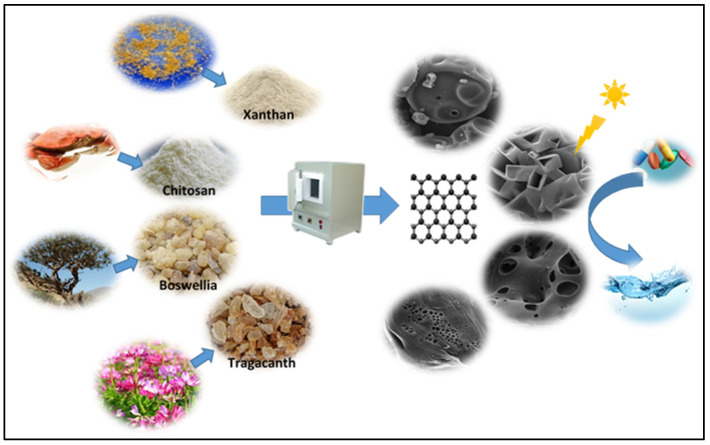
Schematic illustration of synthesis procedure.

A schematic illustration of the synthesis procedure and their catalytic properties is displayed in Scheme 1.

## 4. Conclusions

In this study, bio-graphenes as metal-free, green, and inexpensive catalysts were synthesized with different morphology and porosity via a green method, using natural precursors (Xanthan, Chitosan, Boswellia, Tragacanth gums). These natural polymers act as templates and create special networks that form the 3D graphene structure with special morphology. In this method, the elements present in the natural biopolymers (N) or in the natural composition (P, S) are easily placed in the graphene structure and doped graphenes are synthesized. Doping can improve the catalytic activity due to the structural defects, surface charges, increased electron transfers, and high absorption. The results show that the best catalytic performance is related to the hollow cubic Chitosan-derived graphene, which is due to the doping of N, S, and P to the graphene structure. The Boswellia-derived graphene shows the lowest catalytic properties with the highest surface area, indicating the more effective role of doping in the catalytic activity. In this mechanism, O_2_ dissolved in water absorbs onto the positively charged C adjacent to N dopants to produce the oxygenated radicals, which enables the degradation of antibiotic molecules. Light irradiation increases the amounts of radicals and the rate of antibiotic removal. Oxygen also can accelerate the degradation process via increasing the oxygenated radicals. The quenching tests and continues recovery of doped graphene also confirm the degradation of antibiotics via oxidation process. 

## Data Availability

Data are available within the paper. Should any raw data files be needed in another format, they are available from the corresponding author upon reasonable request.

## Sample Availability

Samples are available from the authors.
